# Measuring 24‐h use of time in people with a diabetes‐related foot ulcer: A feasibility study

**DOI:** 10.1002/jfa2.12045

**Published:** 2024-07-30

**Authors:** Andrew Murphy, Kristin Graham, Timothy Olds, Cathy Loughry, François Fraysse, Dot Dumuid, Ty Stanford, Lisa Matricciani

**Affiliations:** ^1^ Allied Health and Human Performance (AHHP) University of South Australia Adelaide South Australia Australia; ^2^ Department of Podiatry Central Adelaide Local Health Network Adelaide South Australia Australia; ^3^ Innovation, Implementation and Clinical Translation in Health (IIMPACT) University of South Australia Adelaide South Australia Australia; ^4^ Alliance for Research in Exercise Nutrition and Activity (ARENA) University of South Australia Adelaide South Australia Australia; ^5^ Clinical & Health Sciences University of South Australia Adelaide South Australia Australia

**Keywords:** diabetes, diabetes mellitus, diabetes‐related foot ulcer, physical activity, sedentary behaviour

## Abstract

**Background:**

Physical activity (PA), sleep and sedentary time are now recognised as mutually exclusive and exhaustive parts of the 24‐h day—if PA decreases, time spent sleeping, being sedentary or both must increase so that all components equate to 24 h. Recent advances in time‐use epidemiology suggest that we should not consider time‐use domains (PA, sleep and sedentary time) in isolation from each other, but in terms of a *composition*—the mix of time‐use domains across the 24‐h day. While interrelated daily activities are known to be important in the management of diabetes mellitus, few studies have investigated the interrelated daily activities in people with an active diabetes‐related foot ulcer (DFU) and their impact on important outcomes such as wound severity, blood glucose control and health‐related quality of life (HRQoL). This feasibility study aims to determine the acceptability and practicality of measuring 24‐h use of time data in people with a DFU and its associations on important outcome measures for this population.

**Methods:**

Participants wore a wrist‐worn accelerometer for two weeks and completed demographic and HRQoL questionnaires. Outcomes were participant engagement, reported levels of study burden and value and compositional data analysis as a methodological approach for evaluating 24‐h use of time data.

**Results:**

Twenty‐six participants reported low levels of study burden and rated the study value highly. The protocol appears feasible in terms of recruitment (81%) and retention rate (86%). On average, participants were relatively sedentary spending 747, 172 and 18 min in sedentary time, light physical activity and moderate‐to‐vigorous activity, respectively. Sleep appeared adequate with participants obtaining an average of 485 min, but quality of sleep was notably poor with average sleep efficiency of 75%. Compositional data analysis was able to quantify the integrated associations of 24‐h use of time with HRQoL.

**Conclusion:**

The protocol provides an acceptable method to collect 24‐h use of time data in people with a DFU. Efforts to consider and analyse PA as part of a 24‐h activity composition may provide holistic and realistic understandings of PA in this clinical population.

## 1 INTRODUCTION

Diabetes‐related foot ulcers (DFUs) are among the most serious and costly complications of diabetes mellitus (DM) [1]. Approximately 15%–25% of people with DM develop a DFU in their lifetime [1]. Despite appropriate management, ulcers may take months to heal, with 70% of ulcers still active after 20 weeks [2] and recurrence rates are high with 34%, 61% and 70% recurring after one, three and five years, respectively [3]. People with a DFU experience functional declines, reduced health‐related quality of life (HRQoL) and poorer cardiometabolic health when compared to those without a DFU [1, 4]. Concerningly, the 5‐year mortality rate for people with a DFU is 40%―worse than breast cancer [5]—which highlights a need to improve clinical outcomes and reduce disease burden.

Physical activity (PA) is central to diabetes management, with guidelines recommending 150 min/week of moderate‐to‐vigorous physical activity (MVPA) intensity to improve health‐related outcomes [6, 7]. Increased PA has been associated with improved HRQoL, glycaemic control and overall cardiometabolic health [8], while a lack of PA has been reported as an independent risk factor for developing a DFU [9]. Moreover, a recent synthesis of systematic reviews [10] found that PA may be more effective than psychotherapy and pharmacotherapy in treating determinants of HRQoL, including depression, anxiety and psychological stress—common for people with a DFU [11].

Current DFU management guidelines focus on wound management, revascularisation, infection control and offloading [12]. In contrast to recommendations for diabetes management, to protect the wound site and reduce plantar pressures, patients are often encouraged to reduce weight‐bearing activities as it is thought that it may impede wound healing [6, 12]. This can result in patients being inactive for extended periods of time, posing a concern for overall diabetes management and HRQoL. However, the impact of PA on foot ulcer size and wound healing remains contentious, with a recent scoping review suggesting a weak inverse relationship between weight‐bearing PA and ulcer healing [13]. Recent guidelines [14] noted that a reduction in weight‐bearing PA was beneficial for wound healing and health‐related outcomes based on one scoping review [13] and expert opinion, acknowledging a paucity of data.

Despite the known health benefits of PA for people with DM [6], few studies have examined PA in people with an active DFU [15]. Currently, available studies on people with a DFU estimate PA through self‐reported measures such as the International Physical Activity Questionnaire [16, 17]. While questionnaires provide a cost‐effective way to estimate PA, a recent systematic review and meta‐analysis reported low‐to‐moderate validity (*r*_*w*_ = 0.13 to 0.48) when compared to objectively measured estimates [18].

Of the few studies using device‐derived estimates of PA, most have relied on pedometers to record steps/day [19, 20, 21]. Although pedometers provide a more objective method of measuring PA when compared to self‐report measures, they only account for weight‐bearing PA and are sometimes embedded into offloading devices that are seldom worn [19]. Other studies have assessed PA in terms of prescribed exercise programs and their impact on wound healing rates [22, 23]. However, these studies also do not reflect total daily amounts and intensities of PA.

Accelerometers are small devices that are typically worn on the wrist or hip and can provide objective estimates of daily minutes spent in various intensities of PA. In addition to PA, these devices can measure multiple time‐use domains, such as sleep and sedentary time, facilitating the recording of activities over a 24‐h spectrum. Given that sleep and sedentary time are also recognised as important for health and well‐being in people with DM [9, 24], the advantages of measuring multiple time‐use domains via accelerometers may be warranted. Despite these advantages, to our best knowledge, no previous studies have examined 24‐h use of time in people with an active DFU.

Physical activity, sleep and sedentary time are now recognised as mutually exclusive and exhaustive parts of the 24‐h day [25]—if PA decreases, time spent sleeping, being sedentary or both must increase. As a result, recent advances in time‐use epidemiology suggest that we should not consider time‐use domains (PA, sleep and sedentary time) in isolation from each other, but in terms of a time‐use *composition*—the mix of time‐use domains across the 24‐h day [25]. Compositional data analysis (CoDA) is a methodological approach that allows for the analysis of the time‐use composition as it accounts for the relative nature of time‐use domains [25].

Although population‐based studies have investigated the 24‐h time‐use composition [26], efforts to apply this paradigm in clinical populations are lacking. For people with a DFU, this approach may be especially important as they face a dilemma of needing to be physically active (to manage blood glucose control and HRQoL) while also needing to limit weight‐bearing activities to facilitate wound healing. Therefore, different reallocations of time may yield different health outcomes in people with a DFU, but this has yet to be investigated.

To advance this area of research, efforts are needed to objectively measure 24‐h use of time in people with a DFU. This research agenda was recently highlighted in a Delphi study of Australian stakeholders identifying priorities for diabetes‐related research, emphasising the efficacy and safety of exercise in people with diabetes‐related foot complications, while also implementing ‘smart technology’ to help improve DFU healing times [27]. Considering the paucity of available PA data [13], a failure to recognise the compositional nature of time and a lack of device‐derived measures [27], a study protocol capable of addressing these concerns is required.

The primary aim of this feasibility study was to evaluate an approach to collect and analyse device‐derived 24‐h use of time data in people with a DFU. The objectives of this study were to determine as follows:

(1) participant acceptability of a study protocol that required 24‐h activity monitoring;
(2) the practicality for researchers undertaking a study that collects 24‐h use of time, questionnaire and medical record data and
(3) the implementation of CoDA to determine how reallocations of time are associated with health outcomes in people with a DFU.

## 2 METHODS

This study was undertaken at two tertiary metropolitan hospitals in Adelaide, South Australia between November 2022 and April 2023. Ethical approval was granted through the Central Adelaide Local Health Network (CALHN) Human Research Ethics Committee (reference #16937) and University of South Australia's Human Research Ethics Committee (reference #205004).

### 2.1 Participants

A non‐probability purposive sampling method was used to recruit adult participants from inpatient and outpatient settings. Treating podiatrists screened and invited participants to take part in the study if they had an active DFU. Participants were excluded if they had a previous amputation above the talocrural joint, were unable to complete study questionnaires due to physical or cognitive impairments or required a 4‐wheel walker or wheelchair (due to their influence on wrist‐worn accelerometers).

A member of the research team then provided participants with further study details and attained written, informed consent to partake in the study, which involved three aspects: completing a questionnaire, allowing their treating podiatrist to access and pass medical record information to the research team and for them to wear a wrist‐worn accelerometer. Participants could select which of these three study aspects they took part in so that researchers could gauge acceptability levels.

As this was a feasibility study, a sample size calculation was not performed. An anticipated sample size of 30 was considered suitable to provide detail about the acceptability and practicality of the study protocol, based on previous feasibility studies involving people with a DFU [16, 23].

#### 2.1.1 Questionnaire

A questionnaire was administered to participants to complete via an iPad on the day of recruitment. The questionnaire included demographic details such as age, sex, education level, marital status and questions relating to medical history including duration of diabetes, comorbidities, use of an offloading device and last recorded Haemoglobin A1C (HbA1c) measure. The 12‐Item Short‐Form Survey (SF‐12), a HRQoL survey, was also included in the questionnaire. The SF‐12 has been shown to be valid and reliable in assessing HRQoL in people with diabetes [28]. The SF‐12 score was delineated into its mental component score (MCS‐12) and physical component score (PCS‐12). Scores were calculated and normalised based on previous algorithms using Australian population weights [29] and were processed using R (version 4.2.2) [30] in RStudio. Descriptive statistics were generated for both demographic and health‐related measures.

#### 2.1.2 Access to medical records

Medical records were accessed to determine foot ulcer severity, blood glucose control, diabetes type and duration. These variables were selected as they are suitable covariates for analyses that consider the relationship between use of time and health outcomes in people with a DFU [31]. The Wound, Ischemia foot Infection (WIfI) score reported by the treating podiatrist on the date of participant recruitment was collected to determine foot ulcer severity. The WIfI score has been validated for its predictive capabilities in determining the 1‐year risk of major amputation and wound healing times [32]. Blood glucose control was assessed using the participant's most recent HbA1c biomarker. The measure was chosen due to its association with long‐term diabetes complications [33].

#### 2.1.3 24‐h activity monitoring

Participants were fitted with a GENEActiv (Activinsights, UK) accelerometer to their non‐dominant wrist and were instructed to wear the monitor continuously for two weeks, removing only in instances of complete water submersion (i.e., bathing or swimming). Participants were also asked to complete a daily self‐report activity record (activity card) to indicate sleep and wake times, reasons for monitor non‐wear, quality of previous night's sleep and level of daily offloading device adherence (i.e., how often they wore their prescribed offloading device during waking hours).

If participants failed to return their activity card, they were contacted to report approximate sleep and wake times and sleep quality. Two weeks' wear duration was chosen to coincide with the frequency of podiatry visits and to reduce participant burden. If a participant did not have an appointment scheduled for the subsequent two weeks, they were provided a reply‐paid envelope to return the monitor and activity card after 14 days of wear‐time.

The accelerometer was used to objectively measure PA, sleep and sedentary time. While there is no current gold standard to measure 24‐h use of time, the GENEActiv (Activinsights, UK) has previously been used in population‐based studies [34] and has demonstrated excellent technical validity and reliability for classifying different intensities of PA [35, 36] and sleep duration [37] in adults. Concurrent validity has been demonstrated for measures of PA, sleep and sedentary time against other devices, with intraclass correlation coefficient values ranging from 0.82 to 0.95 across multiple time‐use domains [38].

Monitors were calibrated to record data at 50 Hz for 14 consecutive days, commencing at midnight for outpatients on the day of dispense and at midnight on the day of discharge from hospital for inpatients. Raw acceleration data were downloaded and converted into 60‐s epochs using the GENEActiv PC Software (version 3.3) (Activinsights, UK). Epochs were imported into a MATLAB‐based customised software program (*Cobra*) where activity cards, visual inspection of accelerometer traces and the van Hees algorithm [37] were used to identify instances of non‐wear, sleep period time (defined as the difference between sleep onset and offset) and sleep efficiency (the percent of time asleep during sleep periods). The total magnitude of acceleration in each 60‐s epoch was calculated and predetermined magnitude cutpoints of 118, 403 and 1131 gravity units per minute (g.min) [35] were used to classify sedentary time, light physical activity (LPA), moderate physical activity and vigorous physical activity, respectively, keeping in line with established cutpoints in adults. Currently, there are no validated cutpoints for people with a DFU.

A single day was considered valid if it included at least 10 h of wear‐time during waking hours and at least 200 min of sleep time, keeping in line with previous research [26, 39]. Participants were included for subsequent analysis if they had at least four valid days (including one weekend day) recorded. Average time spent daily in sedentary time, sleep, LPA and MVPA was weighted at 5:2 weighting for weekdays: weekends to account for behavioural variability established in previous research [36].

#### 2.1.4 Study feasibility

Feasibility was assessed in terms of protocol acceptability, practicality and implementation of CoDA—three areas of focus addressed by feasibility studies [40]. Reporting was guided by the Strengthening the Reporting of Observational Studies in Epidemiology (STROBE) guideline [41].

Acceptability was considered in terms of participant engagement, perceived study value and burden. Participant engagement was defined in terms of the percentage of participants providing written consent to the study questionnaire and granting access to medical records. Insight into perceived study value was examined using the following 11‐point Likert scale question: ‘On a scale of 0 to 10, please rate the value of this study (0 = not valuable and 10 = extremely valuable)’. Participant burden was determined using the following 11‐point Likert scale question: ‘On a scale of 0 to 10, please rate the burden of this study (0 = not very burdensome/required little effort and 10 = highly burdensome/required a lot of effort)’. Participants were also asked to describe whether they would like to see any changes to the study protocol and what, if any, changes could be made to the study to reduce burden.

Participants were contacted via telephone to answer questions regarding study value and burden at least two weeks after returning the accelerometer. A member of the research team not involved with participant recruitment and enrolment conducted acceptability interviews in an effort to reduce social desirability bias.

Practicality of the study protocol was assessed in terms of participant recruitment, retention and safety, monitor non‐wear, data completeness, accelerometer return and data extraction and activity card completion and return. Participant recruitment was determined as the percentage of patients referred to the research team who enrolled in the study. Participant retention was defined as the percentage of participants enrolled in the study who returned complete data for analyses. Participant safety was determined by any serious incident as a direct result of taking part in the study, admission to hospital or death that may undermine free‐living conditions. Monitor non‐wear was assessed as the average of total non‐wear time (minutes/day) in participants who were included in data analyses. Data completeness was assessed in terms of questionnaire and medical records ascertaining reliable demographic and health‐related data. Accelerometer return and data extraction were expressed as the percentage of monitors that were successfully returned and had data successfully downloaded. Activity card completion and return were assessed in terms of the percentage of activity cards completed and returned to the research team.

Compositional data analysis was used to determine the relationship between use of time reallocations and HRQoL [42]. While there are many outcome measures that could be assessed, HRQoL was considered for this study because it encompasses a variety of health and well‐being constructs. Compositional data analyses were performed in R using “compositions” [43] and “zCompositions” [44] packages. The 24‐h activity composition was created using 5:2 weekday: weekend day weighted sleep duration, sedentary time, LPA and MVPA. The centre of the composition was described by the compositional mean, which is calculated as the geometric mean of each time‐use domain, linearly adjusted so that all parts sum to a total of 1440 (minutes in a 24‐h period). The composition was expressed as four sets of three isometric log ratio (ilr) coordinates following the methods of Chastin and colleagues [42]. As zero values cannot be log transformed, any zeros within MVPA were replaced with small values using the expectation maximisation algorithm [45] from the “zCompositions” package.

Multiple linear regression models (one for each of the four sets of ilrs) were used to determine the association between the 24‐h activity composition and HRQoL components (PCS‐12 and MCS‐12). The estimated regression coefficient for the first ilr coordinate of each model was examined for direction and strength in the outcome when increasing one activity, relative to remaining activities. The multiple linear regression model assumptions of linearity, homoscedasticity and normality were checked via diagnostic plots.

Isotemporal substitution modelling using *one‐for‐remaining* reallocations was performed to examine the model estimated changes in the outcomes when reallocating time in sleep for LPA, MVPA and sedentary time [46]. The R package “codaredistlm” [47] was used to plot isotemporal substitution estimates and associated 95% confidence intervals. Effect sizes (*f* ^2^ values) from compositional data analyses were used in power calculations to determine required sample size for future definitive studies [48]. The Rpackage “pwr” [49] was used for the power analyses.

## 3 RESULTS

**FIGURE 1:**
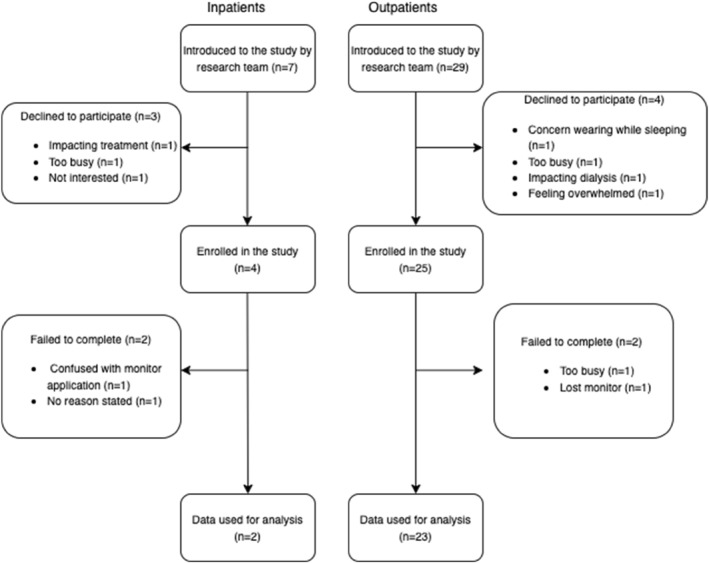
Participant recruitment and retention flow chart.

A total of 29 participants were recruited for the study, with 25 participants returning valid data for analyses (Figure 1). Participants were on average 68.4 (SD = 9.2) years of age, 92% were male, were in a relationship (married or de facto) and had low levels of education. Most participants had type 2 diabetes (92%) with an average diagnosis length of 20 (SD = 11) years, an average HbA1c of 7.9% (SD = 1.8) and between three and six comorbidities. Foot ulcer severity was evenly distributed in terms of 1‐year amputation risk and most participants wore a removable knee‐high cast walker (60%).

The mean PCS‐12 and MCS‐12 (values of the SF‐12) were 35.5 (SD = 6.9) and 49.1 (SD = 12.2), respectively. On average, participants were relatively sedentary spending 747, 172 and 18 min in sedentary time, LPA and MVPA, respectively. MVPA had a skewed distribution, with a median value of 13 (IQR: 5 to 23) and 60% of participants achieving less than 15 min of physical activity, on average, per day. Sleep appeared adequate with participants obtaining an average 485 min, but quality was notably poor, with average sleep efficiency of 75%. The average daily non‐wear time was 18 min. Summary data of participant characteristics are presented in Table 1. Compositional means used for analysis, along with arithmetic means linearly adjusted and unadjusted for non‐wear are reported in Table 2.

**TABLE 1:** Participant characteristics.

Variables	*n* = 25
Age–years	68.4 (9.2)
Gender–count (%)	
Male	23 (92%)
Marital status–count (%)	
Married or de facto	15 (60%)
Widowed	2 (8%)
Divorced	4 (16%)
Never married	4 (16%)
Level of education–count (%)	
Year 12 or below	15 (60%)
Certificate III/IV or trade or Diploma	6 (24%)
Bachelor degree or higher	4 (16%)
SF‐12 score–count	
PCS‐12	35.5 (6.9)
MCS‐12	49.1 (12.2)

Abbreviations: MCS‐12, mental component score; PCS‐12, physical component score; SF‐12, 12‐Item Short‐Form Survey.

**TABLE 2:** 24‐h time‐use domains of participants included for analysis.

24‐h time‐use domains (arithmetic mean)	Arithmetic mean (with non‐wear time)	Arithmetic mean (proportionally imputing non‐wear time)	Compositional mean (proportionally imputing non‐wear time)
Sleep duration (min/d)	484.6	490.5	508.3
Sedentary time (min/d)	747.1	756.6	778.3
LPA (min/d)	172.4	175.1	145
MVPA (min/d)	17.5	17.9	8.4
Monitor non‐wear (min/d)	18.4	―	―

*Note*: Values for compositional means are provided as log ratio values where the total sum represents 1440 min (24‐h day). Average 24‐h time‐use domains are weighted 5:2 (weekday: weekend).

Abbreviations: LPA, light physical activity; MVPA, moderate‐to‐vigorous physical activity.

### 3.1 Acceptability

All enrolled participants (*n* = 29) provided consent to complete a questionnaire, have researchers access specified medical record information and agree to wear an accelerometer. Of the 29 participants who took part in at least one aspect of the study, 26 participants provided feedback to the research team. In terms of acceptability, participants perceived the study protocol to have a low burden with a median score of 1.0 (IQR: 0 to 2) on an 11‐point Likert scale with 0 indicating ‘not very burdensome/required little effort’. Most perceived it to be of high value with a median score of 8.0 (IQR: 6.5 to 10) on an 11‐point Likert scale with 10 indicating ‘extremely valuable’. No recurring suggestions to change the study protocol were made; however, one participant suggested that an incentive (a cup of coffee) would improve the study protocol and two participants were interested in learning more about the accelerometers. Participants provided no clear suggestions for reducing burden in future studies.

### 3.2 Practicality

Data were collected on 16 separate days (3 days for inpatients and 13 for outpatients) by the principal researcher. During this time, 36 patients (seven inpatients and 29 outpatients) were referred by treating podiatrists to take part in the study and 29 enrolled in the study. Prospective participants declined to participate in the study for reasons including being too busy to take part (*n* = 2), currently feeling overwhelmed with their diagnosis (*n* = 1), concerns with getting consistent sleep while wearing a monitor (*n* = 1), concerns with the monitor impacting their treatment (*n* = 2) and not being interested (*n* = 1).

Participant compliance was high (86.2%), with 25 of the 29 participants returning valid time‐use data that satisfied inclusion criteria for analyses. The four participants with invalid time‐use data did not wear their monitor at all, with reasons outlined in Figure 1. Participants complied with the study protocol to wear the monitor continuously and limit non‐wear for activities that required water submersion, with an average non‐wear time of 18 min per day. No participant experienced any serious adverse event related to the study. One participant did report a skin irritation from wearing the accelerometer, but this was self‐limiting and the participant provided valid time‐use data for analyses. A summary of participant recruitment and retention has been outlined in Figure 1.

Data completeness was excellent with 100% of participants completing the questionnaire. In reviewing data, it was noted that three participants were unable to remember their most recent HbA1c result and did not have medical records indicating previous results. Moreover, the average lag from the most recent HbA1c result to accelerometer allocation was considerable, with a median gap of 219 days (IQR: 142 to 354). The activity card was returned by 20 participants. Nineteen participants entered sleep and wake times, 17 participants completed the subjective offloading adherence component, while 10 participants described instances of non‐wear, ranging from ‘dishes’, ‘bathing’ and ‘MRI’. A summary of acceptability and practicality results has been noted in Table 3.

**TABLE 3:** Feasibility measure results.

Feasibility measure	Outcome
Acceptability	
Study burden (median, IQR)	1.0 (0–2)
Study value (median, IQR)	8.0 (6.5–10)
Practicality	
Participant recruitment (%)	80.6
Participant retention (%)	86.2
Monitor non‐wear time (min)	18.4
Activity card return (%)	80
Monitor extraction (%)	100
Monitor return (%)	96

*Note*: Study burden: 11‐point Likert scale (0 = not very burdensome/required little effort and 10 = highly burdensome/required a lot of effort); study value: 11‐point Likert scale (0 = not valuable and 10 = extremely valuable); participant recruitment: number of people invited to take part/number of people who provided consent to participate in the study; participant retention: number of people who consented to participate in study/number of participants who completed the study; activity card return: number of activity cards returned/total number of activity cards provided to participants; monitor extraction: number of monitors successfully downloading activity data/total amount of monitors returned and monitor return: total amount if monitors returned to research team/total amount of monitors dispensed to participants.

Compositional data analysis was used to explore relationships between time‐use data and HRQoL. As shown in Table 4, none of the 24‐h time‐use domains were significantly associated with the physical and mental component scores of the SF‐12 quality of life measure. For the PCS‐12, to detect a medium effect size (*f* ^2^ = 0.3) with an alpha of 0.05, a power of 80% and 8 predictors (age, sex, education, diabetes duration, foot ulcer severity, three ilrs and intercept) at a similar dropout rate of 14%, at least 57 participants would be needed to be recruited. For the MCS‐12, to detect a medium effect size (*f* ^2^ = 0.2) with an alpha of 0.05, a power of 80% and the same 8 predictors and dropout rate, at least 59 participants would need to be recruited.

**TABLE 4:** The association between 24‐h time‐use domains and HRQoL using compositional data analysis.

Time‐use domain^a^ (*n* = 25)	Beta estimate	Std. Error	*p*‐value
Sleep			
PCS‐12	7.98	5.64	0.172
MCS‐12	8.6	10.01	0.40
Sedentary time			
PCS‐12	−7.07	4.88	0.16
MCS‐12	−5.37	8.67	0.54
LPA			
PCS‐12	−1.74	3.55	0.63
MCS‐12	−7.39	6.3	0.25
MVPA			
PCS‐12	0.83	1.71	0.63
MCS‐12	4.2	3.0	0.18

Abbreviations: LPA, light physical activity; MCS‐12, mental component score; MVPA, moderate‐to‐vigorous physical activity; PCS‐12, physical component score.

^a^ Each time‐use domain is considered relative to the remaining time‐use domains.

**FIGURE 2:**
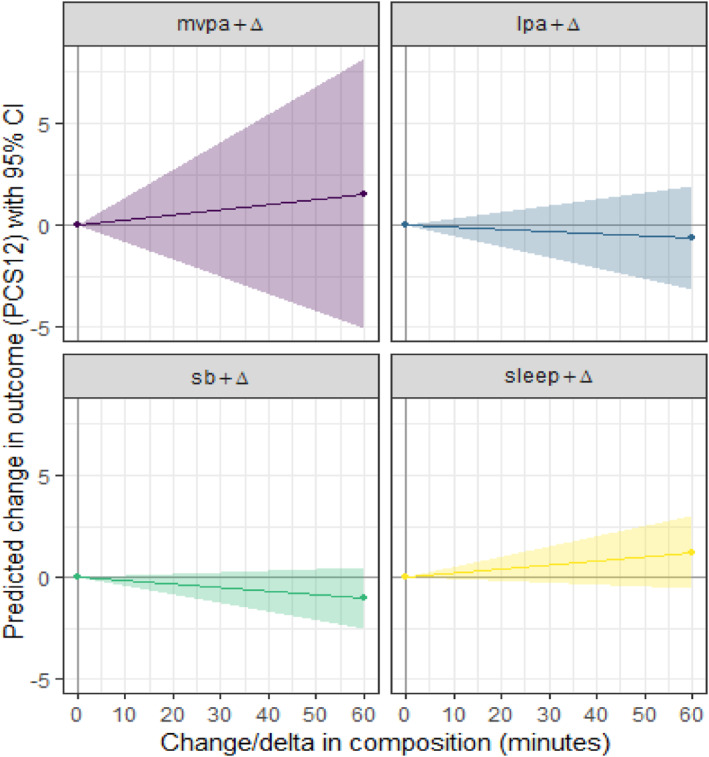
One‐for‐remaining time reallocations for PCS‐12. LPA, light physical activity; MCS‐12, mental component score; MVPA, moderate‐to‐vigorous physical activity; PCS‐12, physical component score. Coloured lines represent the model estimated change in PCS‐12, with surrounding coloured areas indicating 95% confidence intervals.

**FIGURE 3:**
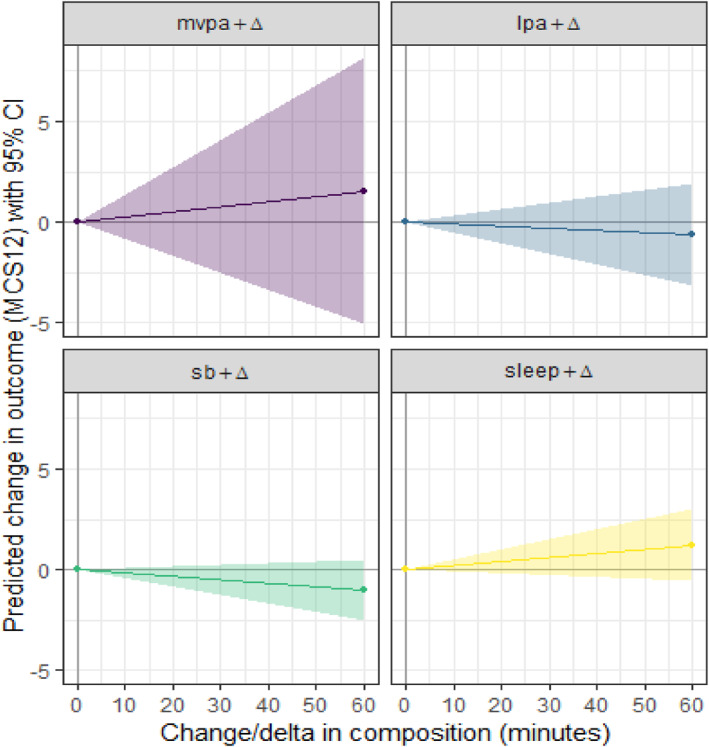
One‐for‐remaining time reallocations for MCS‐12. LPA, light physical activity; MCS‐12, mental component score; MVPA, moderate‐to‐vigorous physical activity; PCS‐12, physical component score. Coloured lines represent the model estimated change in MCS‐12, with surrounding coloured areas indicating 95% confidence intervals.

The beta estimates in Table 4 correspond to the isometric log ratio of the given domain, relative to the geometric mean of the remaining domains. A positive beta estimate indicates a positive association between the isometric log ratio and the outcome. For example, the beta estimate for sleep of 7.98 indicates that a one‐unit increase in the isometric log ratio of sleep versus the remaining domains is associated with *a* +7.98 difference in PCS‐12 score. Figures 2 and 3 present what these beta estimates mean in terms of increases in minutes per day of a particular use of time domain, relative to the remaining domains.

Although not statistically significant, Figures 2 and 3 show that increasing levels of MVPA and sleep tended to be associated with positive differences in both physical and mental component scores, while an increase in sedentary time and LPA tended to be associated with lower component scores.

## 4 DISCUSSION

This feasibility study suggests that it is feasible (in terms of acceptability and practicality) to collect device‐derived 24‐h use of time data in people with a DFU. Compositional data analysis may be a useful methodological approach to better understand the importance of time‐use reallocations for health and well‐being in this clinical population. Overall, participants reported high levels of acceptability, with reports of high study value and low study burden. The study protocol also showed promise with high recruitment (81%) and retention (86%) rates and infrequent adverse events (*n* = 1). Recruitment rates attained in the current study suggest that 14 days of recruiting would be required to recruit 55 participants when using two participant recruiters, which may be of consideration for future, larger studies aiming to reach statistical power.

This study recruited patients from both inpatient and outpatient settings. While enrolled participant numbers differed between inpatients and outpatients, both groups yielded approximately two participants per day of recruitment, suggesting similarity. However, only two of seven inpatients met the required amount of ‘valid days’ for data analyses, which may imply a high attrition rate among inpatients. This may be due to the overwhelming nature of hospital in‐patient stays for people with a DFU as well as previously reported dissatisfaction and mistrust with patient‐provider communication [1, 50]. Overall, recruitment and retention levels in this study were comparable to previous feasibility studies involving participants with a DFU [16] and different clinical populations using wrist‐worn accelerometers [51]. Considering established high mortality and hospitalisation rates in people with a DFU [1], larger longitudinal studies may need to consider how to address data not being recorded in a free‐living environment.

Although excellent questionnaire completion was achieved, missing or dated HbA1c values were recorded. Since blood glucose control is a likely covariate for analyses involving time‐ use and health outcome measures [31], other approaches may be needed to attain more accurate measures in future studies. The GENEActiv (Activinsights, UK) accelerometer was reliable in collecting activity data, with no instances of monitor corruption and only one instance of monitor loss. Monitoring non‐wear throughout the 24‐h day was minimal and similar to population‐based studies using wrist‐worn accelerometers [26]. The activity cards had reduced levels of compliance when compared to monitor adherence, with 80% of participants returning the card. Of those returned, 85% entered self‐reported offloading device adherence levels. Therefore, future protocols may like to consider sending reminders to participants to complete their activity cards or offering incentives for completion. Given the importance of offloading for foot ulcer healing rates [12], future studies may benefit from objectively measuring offloading adherence to accurately use as a covariate.

Compositional data analyses yielded results in the expected direction―higher levels of MVPA and sleep were associated with better quality of life, while higher levels of sedentary time and LPA were associated with poorer quality of life. Although a future, larger study is needed to determine the significance of these associations, it is also important to consider a longitudinal study design to understand the direction of the association. Further, future studies that consider PA in terms of whether an offloading device is in use may be of interest, particularly when examining outcome measures such as foot ulcer severity. Lastly, increasing PA and sleep may be especially challenging in this clinical population where activity may be restricted and sleep is disturbed by pain and worries related to health. Therefore, efforts to understand direct activity trade‐offs (e.g., 1:1 time reallocations) may provide important insight for this population group.

This study found notable differences between objectively measured levels of PA when compared to previous self‐reported estimates in people with a DFU [16, 17]. Recent systematic reviews [18, 52] have raised concerns using self‐report questionnaires, with PA and sedentary time demonstrating low validity and reliability against objective criterion validity. While previous studies that use device‐derived estimates of PA help address concerns with self‐reported measures, they objectively measure steps/day [20, 21] which remains an adjunct contribution to World Health Organisation PA guidelines due to its inability to determine non‐ambulatory PA [7].

Estimates of sedentary time found in the current study are similar to those reported in the only previous accelerometer study in people with a DFU that we are aware of [20]. The current study found participants engaging in numerous extended bouts of sedentary time, with an average bout length of 66 min. Orlando and colleagues [9] in an 8‐year prospective cohort study found that sedentary time had an odds ratio of 2.95 (95% CI: 1.45–6.44) for developing a DFU, with prolonged sedentary time bouts being especially problematic for ulcer development. While high levels of sedentary time may be necessary (to protect the active wound site) [12], Maluf and Mueller [53] suggest a ‘physical stress theory’ where sedentary time may decondition soft tissue adaptations in healthy tissue leading to a decreased capacity of the skin to tolerate stress and increase ulceration risk. This is noteworthy given that an initial DFU is frequently followed by new ulcerations typically occurring on the contralateral foot from the previous/active ulcer site or at different anatomical locations on the same ulcerated foot [54]. This may suggest that sedentary time, while beneficial for acute wounds, may predispose other areas of the feet for future ulcerations.

Sleep remains an under‐studied activity domain in people with a DFU. In contrast to this study, which considers sleep as a potential predictor of poor health and complications, studies that examine sleep in people with a DFU tend to consider poor sleep as a consequence of DFUs [55]. Although most participants in this study met sleep duration guidelines (min/day) [56], only six participants met sleep efficiency (a measure of sleep quality) recommendations [57]. Compositional data analysis considers sleep as a component of time but may also need to account for sleep as a multidimensional construct involving sleep quality.

To our knowledge, this is the first study to examine the feasibility of an approach to collect and analyse 24‐h use of time data in people with a DFU. Although this study provides important insight to help advance this field of research, there are several limitations that also need to be addressed. First, while the cutpoints used for delineating PA have been validated for adults in population‐based studies [35], they have not been validated for people with a DFU. Second, the time spent in LPA and MVPA could not be demarcated into weight‐bearing and non‐weight‐bearing PA, therefore limiting inferences about weight‐bearing PA—which may be important if foot ulcer severity was the outcome measure of interest. Third, clinician perspectives were not considered and levels of study value and burden from a clinician perspective may be valuable for future longitudinal studies. Fourth, while males are more likely to develop a DFU [12], the proportion of males in our study (92%) may limit the generalisability of results given that males typically account for 70% of DFUs [1].

This study suggests that it is feasible (in terms of acceptability and practicality) to collect 24‐h device‐derived use of time data in people with a DFU. Compositional data analysis offers a promising method to better understand how PA and other components of time‐use are associated with health outcomes. Given the high burden of DFUs on both the individual and the broader health system, improving our understanding on how PA and other time‐use domains impact ulcer healing and health‐related outcomes could be valuable and may provide additional targeted strategies for health professionals in the management of DFUs.

## AUTHOR CONTRIBUTIONS

**Andrew Murphy**: Conceptualization; methodology; data curation; writing – original draft; writing – review & editing. **Kristin Graham**: Writing – original draft; review and editing; conceptualization; methodology. **Timothy Olds**: Conceptualization; methodology; writing – review & editing. **Cathy Loughry**: Conceptualization; methodology; writing – review & editing. **Ty Stanford**: Data curation; review and editing. **Lisa Matricciani**: Conceptualization; methodology; data curation; writing – original draft; writing – review & editing.

## CONFLICT OF INTEREST STATEMENT

All the authors declare that they have no conflicts of interest.

## ETHICS STATEMENT

Ethical approval was granted through the Central Adelaide Local Health Network (CALHN) Human Research Ethics Committee (reference #16937) and University of South Australia's Human Research Ethics Committee (reference #205004).

## CONSENT FOR PUBLICATION

Authors obtained written consent for participation and publication including from participants in photographs.

## Data Availability

Raw data are available from authors on request.
